# Single-port laparoscopic surgery for cecum cancer with intussusception: a case report

**DOI:** 10.1186/s40792-024-01962-2

**Published:** 2024-07-03

**Authors:** Yuhei Oshima, Yasuhiro Ishiyama, Hiroto Tanaka, Tadatsugu Fujii, Naoto Okazaki, Toshimasa Ishii, Katuya Deguchi, Yasumitsu Hirano, Isamu Koyama

**Affiliations:** Department of Gastroenterology, Saitama Medical University International Medical Center, Yamane 1397-1, Hidaka, Saitama 350-1298 Japan

**Keywords:** Intussusception, Single-port laparoscopy, Colon cancer

## Abstract

**Background:**

Most adult cases of intussusception are caused by colorectal cancer, and emergency surgery is performed when symptoms such as abdominal pain and vomiting are present. The patient must customarily undergo both bowel decompression and radical surgery for colorectal cancer at the same time, and laparotomy is generally the procedure of choice.

**Case presentation:**

An 86-year-old woman presented to our hospital with diarrhea and bloody stools. Preoperative examination revealed the presence of a cancerous tumor in the advanced part of the transverse colon and bowel intussusception. Radical surgery was successfully performed using the laparoscopic single-port technique through a small incision at the umbilical site to treat intussusception caused by cecum cancer.

**Conclusions:**

With only one wound site at the umbilicus, this single-port laparoscopic approach is much less invasive than endoscopic surgery that requires four to five incision wounds to perform the procedure. Furthermore, the patient was discharged without major complications and this surgical technique could be of great benefit if established as a standard procedure in the future.

## Background

Adult intussusception is relatively rare, accounting for about 1% of intestinal obstructions and 5–10% of all intussusceptions [1–3]. The most common sites of colonic intestinal intussusception are the cecum and the sigmoid colon, and the number of intussusception cases caused by malignant tumors has been increasing recently [3]. Intussusception with abdominal pain and vomiting is a clear indication for emergency surgery [4].

With the widespread use of laparoscopy for a range of diagnostic and independent surgical purposes, there have been sporadic reports of laparoscopic procedures performed on a standby basis for the treatment of adult patients with intussusception [4–6]. In this case, we report the first surgical procedure that involved a single incision laparoscopic ileocecal resection for a patient with intussusception caused by cecum cancer in Japan.

## Case presentation

**Fig. 1:**
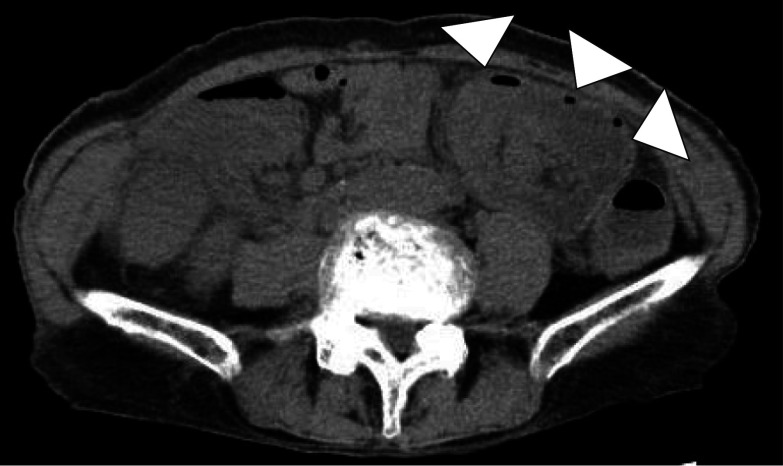
CT image of thorax and abdomen. The lesion was located in the transverse colon and was intussuscepted to the small intestine (arrows)

**Fig. 2:**
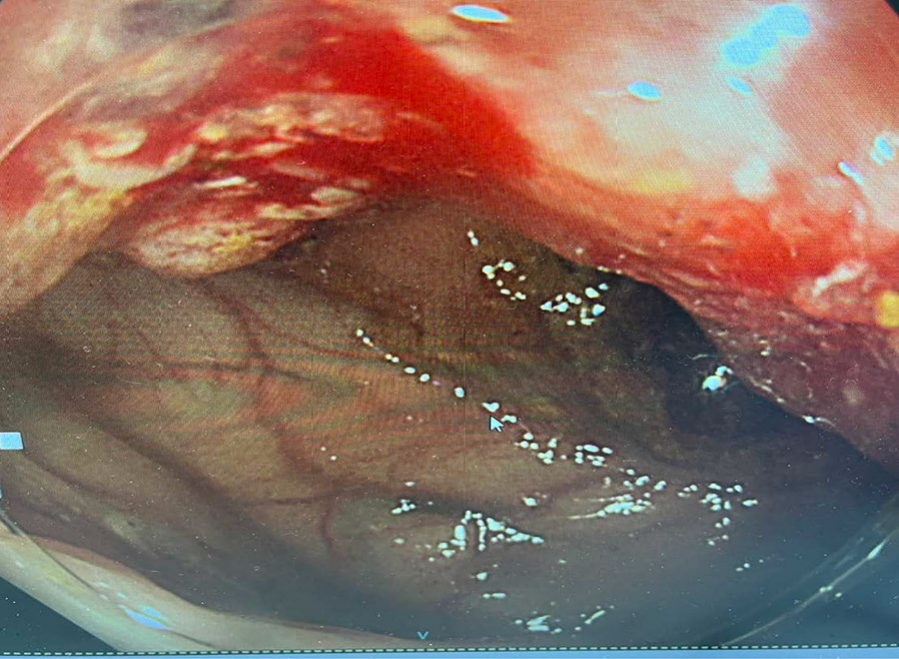
Colonoscopy revealed a 2/3 circumferential Type 2 lesion in the transverse colon just beyond the splenic curvature, which was difficult to pass through the scope

An 86-year-old woman presented to our hospital with diarrhea and bloody stools. Preoperative examination revealed the presence of a tumor in the advanced part of the transverse colon and a bowel accumulation. She had hypertension and bronchial asthma. She was 148.0 cm tall and weighed 39.0 kg. She was conscious and coherent, with no anemia of the eyelid membrane or eyes, and no abdominal symptoms. Laboratory tests were within the normal range, including a carcino-embryonic antigen level of 2.7 ng/mL and a carbohydrate antigen 19-9 level of 11.3 U/mL. Computed tomography showed that the ascending colon on the mouth side appeared to be stuck to the transverse colon, and the patient had intussusception (Fig. 1). Colonoscopy revealed a 2/3 circumferential Type 2 lesion in the transverse colon just beyond the splenic curvature, which was difficult to pass through the scope (Fig. 2). Biopsy results showed Group 5. The patient had no abdominal symptoms such as vomiting or abdominal pain and was stable.

**Fig. 3:**
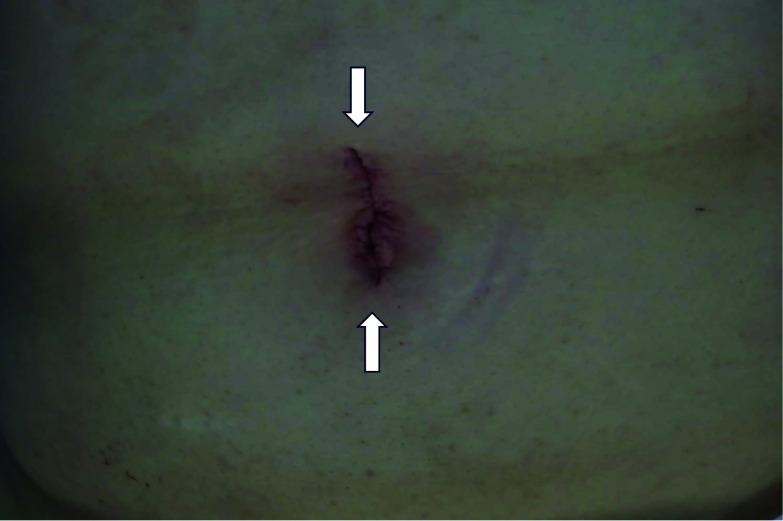
Postoperative image of the umbilical wound, using single-port surgery (arrows)

**Fig. 4:**
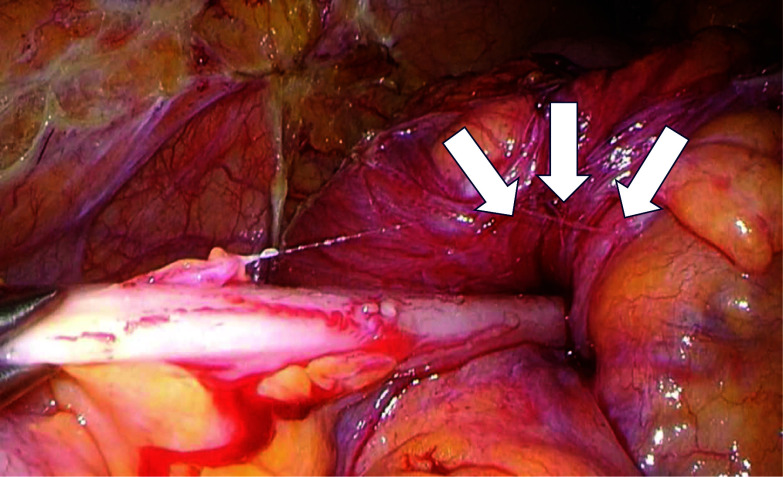
Intraoperative photograph of the patient’s transverse colon, which was found to be intussuscepted and an attempt was made to evacuate the ileocecum manually (arrows)

After observing the intra-abdominal cavity under direct laparoscopic vision through a small incision at the umbilical site (2.5 cm) (see Fig. 3 for postoperative image of wound), an attempt was made to manually evacuate the patient’s ileocecum, which was found to be piled up. The colon was fixed, the mesentery was loosely fixed, and the procedure was performed under direct vision until the colon was released (Fig. 4). The surgical technique was similar to a standard laparoscopic colorectal resection using a flexible scope and standard laparoscopic instruments were used.

Diagnosis of intussusception due to cecum cancer was made at this point. Decompression was first done laparoscopically, and we completed central vascular ligation by dissecting the ileocolic artery and vein using LigaSure™ Maryland (Medtronic) vessels at their origins. The specimen was then extracted through the umbilical incision. The operative time was 126 min, and blood loss was 3 mL.

**Fig. 5:**
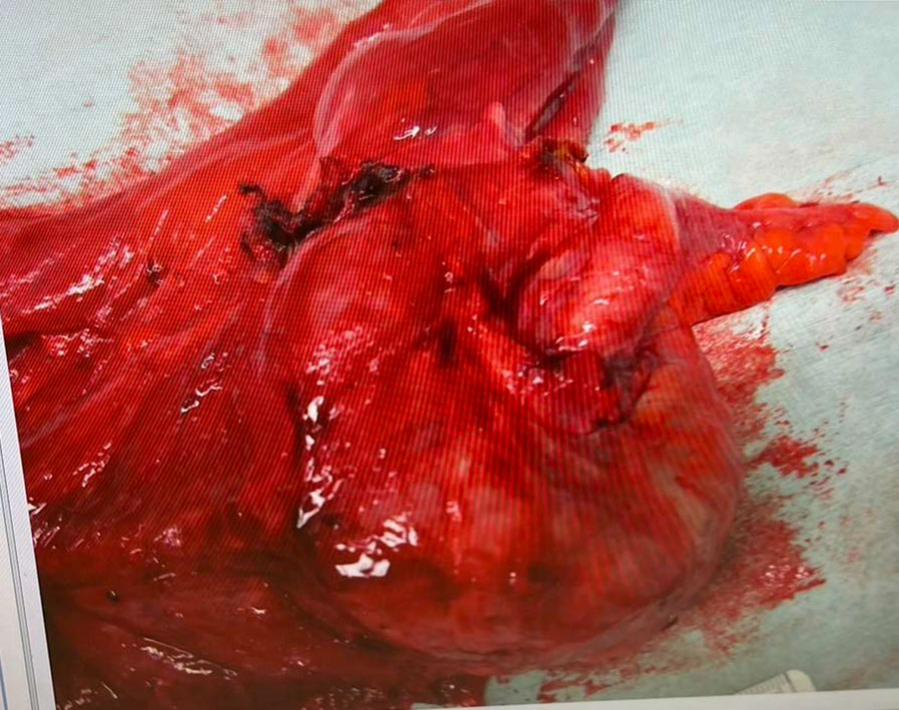
Pathological image of tumor, which was a type 1 lesion. No lymph node metastasis or distant metastasis was observed

The postoperative course was good without any complications, and the patient was discharged 9 days after surgery. Postoperative pain was managed with twice-daily oral intake of Kalonar, without any need for epidural anesthesia. Histopathologically, the surgical margin was negative for cancer, and the tumor was diagnosed as adenocarcinoma pT2N0M0 stage I (TNM classification) (Fig. 5). One year after surgery, the patient was alive without any evidence of recurrence.

## Discussion

In children, the majority of cases are idiopathic, whereas in adults, nearly 90% of cases of intussusception are due to an identifiable etiology [4, 5]. In general, the majority of intussusception cases are secondary to benign lesions. Malignant etiologies account for only 30% of lesions and include primary (mainly adenocarcinoma) or metastatic tumors [4, 5]. If the cause of the lesion is benign, there is no objection to preoperative restoration and minimal resection of the intestinal tract. However, if the cause of the lesion is malignant, there is often debate as to whether restoration should be performed or not. The main reason for preoperative and intraoperative reconstruction is to minimize excessive bowel resection and postoperative digestive and absorptive dysfunction [6].

On the other hand, the most compelling reasons for not performing laparoscopic restoration is the risk of inducing intraperitoneal dissemination of malignant cells, venous embolization, and hematogenous metastasis. Furthermore, the risk of perforation is increased by forcibly adjusting an intussuscepted intestine with inflammation and edema [7]. In recent years, preoperative decompression is a preferred treatment to avoid emergency surgery for cancer. Some researchers have also suggested that decompression can reduce the time for preoperative preparation of the bowel and careful radiographic and endoscopic examination can detect strangulated intussusception that cannot be resected preoperatively [8].

In terms of surgical techniques for intussusception, Jamshidi et al. compared the postoperative complications of laparoscopic surgery (LS) and open surgery (OS) in patients with surgical bowel intussusception. They note that LS is customarily used a diagnostic tool to determine the need for OS, which decreases the incidence of OS and its complications [9]. In addition, Kang et al. state that LS may cause adult bowel complications, and even more serious intraoperative complications than OS [10].

This is the first case report of a single-port laparoscopic surgery for intussusception caused by a malignant tumor in Japan. In our hospital, single-port laparoscopic surgery is the first choice for patients with right-sided colon cancer on the basis that no complications have also been reported regarding the long-term results of single-port surgery in patients with colon cancer [11]. The advantages of single-port surgery include superior cosmetic appearance and less postoperative pain [12]. For these reasons, single-port surgery was the preferred option even for this case of intussusception caused by colorectal cancer. Laparoscopic repair of intussusception is often difficult by having to push the intestine out from the anal side and requires delicate traction of the oral side of the intestine with intestinal forceps. If laparoscopic repair is not successful, the patient should be treated with an open laparotomy, which is more invasive.

For this particular case, the intussusception was observed through a small incision in the umbilical region, and after confirming that there was no problem with the intestinal characteristics, a gentle manual manipulation was performed. There was an advantage in having a prior umbilical incision for this single incision laparoscopic surgery because the right colon was long and could be removed through the umbilical incision after removing the lateral attachment. Another advantage of single incision laparoscopic surgery is that conversion to open surgery can be easily performed if it is deemed necessary. After successful decompression for intussusception in extracorporeal from the umbilical incision, a single-port laparoscopic resection of the ileocecal area with D3 dissection was completed.

## Conclusion

This case demonstrates that the single-port laparoscopic approach is a less invasive and successful surgical technique for the treatment of intussusception due to colon cancer, which is preceded by a small laparotomy that facilitates observation of the state of intussusception and decompression. However, we should not hesitate to add a port or to perform an open transition when necessary and we believe that additional studies of comparable intussusception surgery cases are needed to further verify our findings.

## Data Availability

The data in this study are available from the corresponding author upon reasonable request.

## Abbreviations

LS: Laparoscopic surgery
OS: Open surgery
